# Immune Landscape Refines the Classification of Colorectal Cancer With Heterogeneous Prognosis, Tumor Microenvironment and Distinct Sensitivity to Frontline Therapies

**DOI:** 10.3389/fcell.2021.784199

**Published:** 2022-01-10

**Authors:** Zaoqu Liu, Yaxin Guo, Xiuxiu Yang, Chen Chen, Dandan Fan, Xiaoke Wu, Chaohua Si, Yanxin Xu, Bo shao, Zhuang Chen, Qin Dang, Wenming Cui, Xinwei Han, Zhenyu Ji, Zhenqiang Sun

**Affiliations:** ^1^ Department of Colorectal Surgery, The First Affiliated Hospital of Zhengzhou University, Zhengzhou, China; ^2^ Department of Interventional Radiology, The First Affiliated Hospital of Zhengzhou University, Zhengzhou, China; ^3^ School of Basic Medical Sciences, Zhengzhou University, Zhengzhou, China; ^4^ Academy of Medical Sciences, Zhengzhou University, Zhengzhou, China; ^5^ Henan Institute of Medical and Pharmaceutical Sciences, Zhengzhou University, Zhengzhou, China; ^6^ Department of Hematology, Tongji Hospital, Tongji Medical College, Huazhong University of Science and Technology, Wuhan, China; ^7^ School of Life Sciences, Zhengzhou University, Zhengzhou, China; ^8^ Department of Neurology, The First Affiliated Hospital of Zhengzhou University, Zhengzhou, China

**Keywords:** colorectal cancer, genomic alteration, mutational signature, molecular subtype, prognosis, metastasis

## Abstract

The immune microenvironment has profound impacts on the initiation and progression of colorectal cancer (CRC). Therefore, the goal of this article is to identify two robust immune subtypes in CRC, further provide novel insights for the underlying mechanisms and clinical management. In this study, two CRC immune subtypes were identified using the consensus clustering of immune-related gene expression profiles in the *meta*-GEO dataset (n = 1,198), and their reproducibility was further verified in the TCGA-CRC dataset (n = 638). Subsequently, we characterized the immune escape mechanisms, gene alterations, and clinical features of two immune subtypes. Cluster 1 (C1) was defined as the “immune cold subtype” with immune cell depletion and deficiency, while cluster 2 (C2) was designed as the “immune hot subtype”, with abundant immune cell infiltration and matrix activation. We also underlined the potential immune escape mechanisms: lack of MHC molecules and defective tumor antigen presentation capacity in C1, increased immunosuppressive molecules in C2. The prognosis and sensitivity to 5-FU, Cisplatin and immunotherapy differed between two subtypes. According to the two immune subtypes, we developed a prognosis associated risk score (PARS) with the accurate performance for predicting the prognosis. Additionally, two nomograms for overall survival (OS) and disease-free survival (DFS) were further constructed to facilitate clinical management. Overall, our research provides new references and insights for understanding and refining the CRC.

## Introduction

Colorectal cancer (CRC) is a malignant tumor that originates from resident somatic stem cells and colorectal epithelial tissue (Perekatt et al., 2018). According to the anatomical location, CRC can be divided into colon cancer and rectal cancer. Adenocarcinoma is the most common pathological type of CRC, and very few are squamous cell carcinoma. Currently, the clinical staging system of CRC based on histopathology and medical imaging has limited ability in the clinical management of CRC (Benson et al., 2018; Weiser, 2018). Recently, the molecular classification improved the staging system and provides clues for mining CRC treatment targets (Calon et al., 2015). However, these molecular classification studies were primarily focused on tumor cell-intrinsic characteristics and did not consider the key roles of tumor immunity and tumor microenvironment in tumor progression.

Previous studies reported that the immune system and immune-related genes played vital roles in tumor initiation, progression, prognosis, recurrence, and chemotherapy and immunotherapy benefits (Terzic et al., 2010; Ye et al., 2019; Bruni et al., 2020; Liu et al., 2021c; Liu et al., 2021e). A TCGA-pancancer study conducted extensive immunogenomic analysis and identified six pancancer immune subtypes (PISs): wound healing (PIS1), IFN-gamma dominant (PIS2), inflammatory (PIS3), lymphocyte depleted (PIS4), immunologically quiet (PIS5), and TGF-beta dominant (PIS6), which spans across traditional cancer classifications based on anatomical site of origin and suggests that certain therapeutic approaches may be considered regardless of tumor location and histology (Thorsson et al., 2018).

Recently, immune checkpoint inhibitors (ICIs) have shown amazing therapeutic effects in a variety of tumors (Brahmer et al., 2012; Topalian et al., 2012; Wolchok et al., 2017). However, not all patients could benefit from immunotherapy, which might be due to the involvement of tumor immune escapes. Tumor immune escapes refer to the phenomenon that tumor cells evade recognition and attack by the immune system through a variety of mechanisms, thereby continuing to survive and proliferate. In this study, we aimed to identify two robust immune subtypes with differences in tumor immune escapes, molecular alterations, and clinical outcomes, to further advance the understanding and clinical management of CRC.

## Materials and Methods

### Dataset Source and Preprocessing

Public gene-expression data and full clinical annotation were searched in Gene-Expression Omnibus (GEO) and the Cancer Genome Atlas (TCGA) databases. A total of 1836 patients from eight eligible CRC cohorts including GSE17536 (*n* = 177), GSE17537 (*n* = 55), GSE29621 (*n* = 65), GSE38832 (*n* = 122), GSE39084 (*n* = 70), GSE39582 (*n* = 585), GSE72970 (*n* = 124), and TCGA-CRC (n = 638) were pooled in this study for further analysis. All GEO datasets were from the GPL570 platform. Basic information of datasets included in this study were shown in Supplementary Table S1. The Robust Multi-Array Average algorithm (RMA) algorithm was utilized to normalize the GEO microarrays. The Combat function implemented in the SVA package was applied to remove the batch effects among the GEO datasets (Supplementary Figure S1A,B). The TCGA RNA-seq data was converted into log2 (TPM+1) format. The clinical information, mutation, copy number variant (CNV), and methylation data of TCGA-CRC were downloaded from the TCGA official website. Additionally, we also included three immunotherapy cohorts (Roh cohort, GSE100797, and GSE78220) for subclass mapping (SubMap) analysis (Roh et al., 2017). Complete response (CR) and partial response (PR) were regarded as immunotherapy responders while stable disease (SD) and progressive disease (PD) were regarded as immunotherapy non-responders, and patients who were not evaluable (NE) were removed. All the expression data were further transformed into Z-score.

### Gene Source

A total of 1793 immune-related genes were enrolled from the ImmPort database. A total of 728 immune cell consensus biomarkers were extracted from a previous report (Charoentong et al., 2017). To account for yet unknown immune-related genes, we included genes that were significantly correlated with at least one gene in the meta-GEO cohort. The thresholds were set as the absolute value of Spearman correlation >0.7 and false discovery rate (FDR) < 0.05. Eventually, a total of 2,798 genes were recruited for further analysis in this study (Supplementary Table S2).

### Identification of Immune Subtypes and Gene Modules

Based on 1,198 samples in the meta-GEO cohort, we used the ConsensusClusterPlus package to perform consensus clustering. This procedure was repeated 1,000 times to ensure the stability of classification. The number of clusters K was set to 2–9, and the sampling ratio of the sample was set to 0.8. Unsupervised clustering methods (K-means) were used to identify immune subtypes for further analysis (based on Euclidean distance). To identify immune gene modules, we also applied the consensus clustering using the same settings and parameters. Cumulative distribution function (CDF) and proportion of ambiguous clustering (PAC) were used to identify the optimal K.

### Validation of Immune Subtypes

To further evaluate the reproducibility of the clusters generated from consensus clustering in the meta-GEO cohort, the in-group proportion (IGP) statistical analysis was employed to demonstrate the existence of these clusters in the TCGA-CRC validation cohort. The IGP was defined as the proportion of samples in a group whose nearest neighbors were also in the same group (Kapp and Tibshirani, 2007). We firstly calculated a centroid for each cluster found in the meta-GEO cohort, each sample in the TCGA cohort was classified to a cluster whose centroid had the highest Pearson correlation with a centroid. Later, the clusterRepro package was utilized to perform IGP statistical analysis, and the statistical significance of IGP was assessed with 1,000 permutations (Kapp and Tibshirani, 2007). The *p*-value and IGP statistics were used to estimate cluster quality as in the previous study (Liu et al., 2021b; Liu et al., 2021f).

### Functional Annotation and Immune Cells Infiltration Assessment

The gene-set enrichment analysis (GSEA) was performed between two subtypes, and gene terms with FDR <0.05 were significant. We also applied the gene-set variation analysis (GSVA) to find the specific Hallmark pathways of each subtype. The single sample gene-set enrichment analysis (ssGSEA) algorithm was used to evaluate the infiltration abundance of 30 different types of tumor microenvironment (TME) cells. Considering that fibroblasts and epithelial cells are also important cellular components in TME, thus, in addition to including consensus biomarkers of 28 immune cells, we also included 40 marker genes of fibroblasts and endothelial cells from a previous study (Supplementary Table S3) (Becht et al., 2016).

### Collection and Investigation of Immune Escape Indicators

A series of tumor immune-related indicators (Supplementary Table S4), including stromal and leukocyte fractions, nonsilent mutation rate, neoantigen burden, cancer testis antigens (CTA) score, aneuploidy score, intratumor heterogeneity, number of segments, number or fraction of segments with loss of heterozygosity (LOH), fraction altered, homologous recombination deficiency (HRD), BCR/TCR diversity (Shannon Entropy and Richness) score (Thorsson et al., 2018), microsatellite instability (MSI) score (Bonneville et al., 2017), cytolytic activity (Rooney et al., 2015), antigen processing and presenting machinery score (APS) (Wang et al., 2019) and the expression of immunomodulator molecules (Liu et al., 2021d; Liu et al., 2021h), were enrolled or calculated for the investigation of potential immune escape mechanisms in the four clusters. Moreover, multi-omics regulation of 75 immunomodulator molecules was further analyzed (Supplementary Table S5).

### Genomic Alterations

We used the MutationPattern package to convert the mutation data into a matrix of 96 mutation spectra. Then the NMF package was performed to extract mutation signatures of the two immune subtypes. The MutSigCV algorithm was executed to identify significant mutation genes (SMGs). The screening criteria for frequently mutated genes (FMG) are set to *q* < 0.05 and mutation frequency >10%. GISTIC 2.0 was used to identify chromosome arms or chromosome segments that are significantly amplified or deleted. Segments with *q* < 0.05 and copy number variation frequency >0.3 are considered as driver segments.

### Treatment Prediction for Immune Subtypes

We use the pRRophetic package to predict the sensitivity of the two subtypes to Cisplatin. The sensitivity was quantified by IC50. The lower the value, the stronger the sensitivity. As in previous studies, the TIDE and SubMap algorithms were utilized to predict the response of the two subtypes to immunotherapy (Liu et al., 2021a; Liu et al., 2021e; Liu et al., 2021g).

### Generation of a Prognosis Signature

To identify a prognosis signature for facilitating the clinical management of CRC, we constructed a pipeline. 1) The limma package was utilized to screen differentially expressed genes (DEGs) between C1 and C2 in both meta cohorts and TCGA cohort respectively, and the filtration criteria were adjust-*p* <0.05 and |log_2_ fold change| >1. The overlapping DEGs in both cohorts were defined as consensus DEGs (CDEGs). 2) For the CDEGs expression matrix, we next transformed it into the gene pairs matrix. The gene pair was concerned about the mathematical relationship between the mRNA expression of two genes, and ignored the batch effects of different platforms and facilitated the clinical application. For example, for a gene pair (gene1 and gene2), if the expression of gene1 was greater than gene2 in sample x, the gene pair value in the sample was labeled as 1, otherwise it was labeled as 0. 3) If a gene pair had more than 90% of the same value in all samples, the gene pair was removed. 4) Univariate Cox regression analysis extracted the gene pair with predominant prognostic significance for further analysis (adjust-*p* <0.05 and |HR-1| >0.5; HR: Hazard ratio). 5) The Lasso regression was employed to fit a well-behaved model for predicting overall survival (OS), and the minimal lambda value determined the number of gene pairs and the optimal model. The final model was as follows: risk score = ∑ Value (gene pair) * Coef (gene pair), where Value (gene pair) denoted the value of a gene pair (0/1) and Coef (gene pair) represented its regression coefficient. The risk score was termed prognosis associated risk score (PARS). 6) We calculated the PARS of each patient and performed the Kaplan-Meier survival analysis for OS and DFS. The univariate Cox regression was applied to reveal the prognosis value in various cohorts. The receiver operator characteristic (ROC) curves and Concordance index (C-index) were utilized to assess the performance of PARS in predicting prognosis. 7) In order to ensure the stability of the signature, the process of constructing the model was performed in the *meta*-GEO cohorts, and the TCGA-CRC cohort was used for validation.

### Statistical Analysis

The Fisher’s exact test was used to evaluate the co-occurrence or rejection of FMGs. The Spearman or Pearson correlation analysis was used to calculate the correlation coefficient of two variables. The comparison between the two groups was carried out by Wilcoxon rank sum test, when three or more groups were compared by Kruskal-Wallis test. The Kaplan-Meier method was used to generate survival curves for prognostic analysis, and the log-rank test was used to determine the significance of differences. The univariate Cox regression analysis was used to calculate the hazard ratio (HR) of the variables, and multiple Cox regression was used to determine independent prognostic factors. The ROC curves were analyzed by the timeROC package. The enrichment analysis was performed by the clusterProfiler package. The survminer package was applied to determine the optimal cut-off value of PARS for the Kaplan-Meier survival analysis. The maftools R package was utilized to analyze data and visualize the mutation waterfall plots. All statistical values were tested by two-sided test, and *p* < 0.05 was considered statistically significant.

## Results

### Immune Subtypes and Gene Modules

**FIGURE 1:**
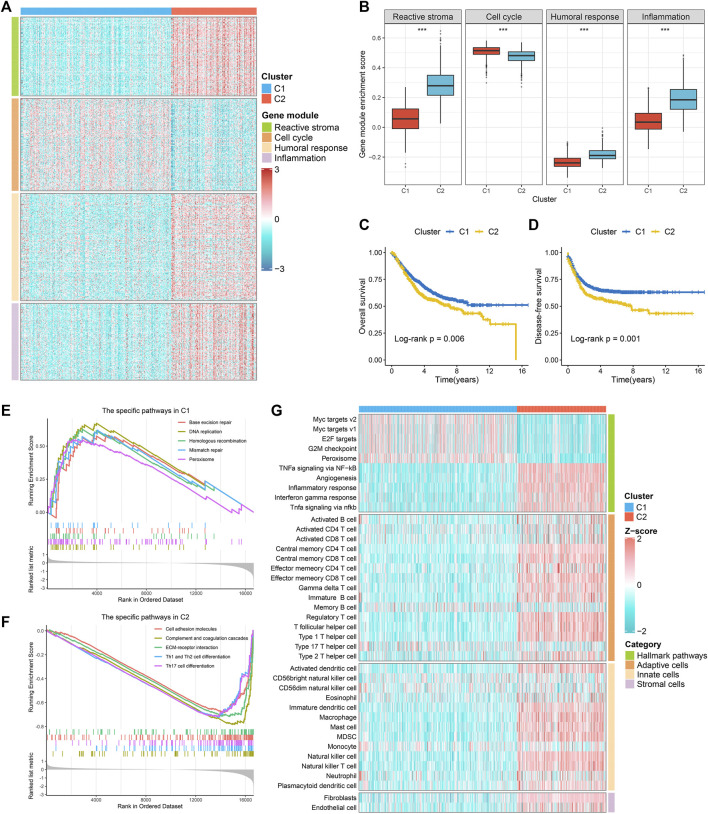
The specific functions and survival status of each subtype in the *meta*-GEO cohort. **(A)** The expression profiles of gene modules between two subtypes. **(B)** The ssGSEA algorithm was performed to quantify the relative abundance of four gene modules between two subtypes. **(C,D)** Kaplan-Meier survival analysis of overall survival **(C)** and disease-free survival **(D)** according to the two subtypes. **(E,F)**. GSEA was performed to identify specific KEGG pathways in C1 **(E)** and C2 **(F)**. **(G)**. The hallmark analysis (GSVA) and immune cell infiltration estimation (ssGSEA) of two subtypes.

Based on the immune-related gene expression profiles, we identified two robust immune subtypes (C1 and C2) in the *meta*-GEO discovery cohort (Suppplementary Figure S1C). The CDF curves and PAC analysis verified the results (Supplementary Figures S1D,E). In the two-dimensional PCA analysis, the spatial distribution contours of the two subtypes basically did not overlap (Supplementary Figure S1F). To ensure the reproducibility and robustness of the immune subtypes derived from the GEO cohort, we further calculated the IGP statistic to validate the immune subtypes in the TCGA-CRC validation cohort. These two immune subtypes were highly consistent between the discovery and validation cohorts, with the corresponding IGP values at 91.3 and 93.7%, respectively (all, *p* < 0.001). In addition, we also identified four gene modules (GM1-4) (Supplementary Figures S2A–C). Enrichment analysis showed that GM1-4 was mainly related to reactive stroma, cell cycle, humoral response, and inflammation, respectively (Supplementary Figure S2D). As shown in Figures 1A,B and Supplementary Figures S3A,B, GM1/3/4 was higher in C2, while GM2 was superior in C1. Overall, C2 was biased towards immune activation and matrix activation, and C1 was biased towards cell proliferation. Survival analysis revealed that C1 had better OS and disease-free survival (DFS) than C2 (Figures 1C,D and Supplementary Figures S3CD). GSEA analysis showed that C2 was mainly enriched in matrix activation and immune activation related pathways (Figure 1E and Supplementary Figure S3E), while C1 was mainly enriched in cell proliferation related pathways (Figure 1F and Supplementary Figure S3F). We further identified the specific Hallmark pathway of each immune subtype. The results were consistent with the GSEA analysis: C2 was mainly related to matrix activation and immune activation, and C1 mainly enriched cell proliferation related pathways (Figure 1G and Supplementary Figure S3G). The ssGSEA algorithm was further used to evaluate the infiltration abundance of TME cells in the training and the validation datasets. It was observed that C1 showed relatively low infiltration of immune cells, while C2 showed high infiltration abundance in most immune cells (Figure 1G and Supplementary Figure S3G). A TCGA-pancancer study proposed six immune clusters: wound healing (PIS1), IFN-gamma dominant (PIS2), inflammatory (PIS3), lymphocyte depleted (PIS4), immunologically quiet (PIS5), and TGF-beta dominant (PIS6) (Thorsson et al., 2018). In TCGA-CRC cohort, the PIS5 was absent, and only five PISs were identified in CRC, predominantly PIS1 (77.1%) and C2 (17.4%) (Soldevilla et al., 2019). In our subtypes, C1 and C2 both had the highest proportion of PIS1, notably PIS1 was more identified in C1, whereas PIS2 was particularly dominant in C2. Of note, there was no PIS6 in C1 (Supplementary Figure S4).

### Exogenous Immune Escape Mechanisms

**FIGURE 2:**
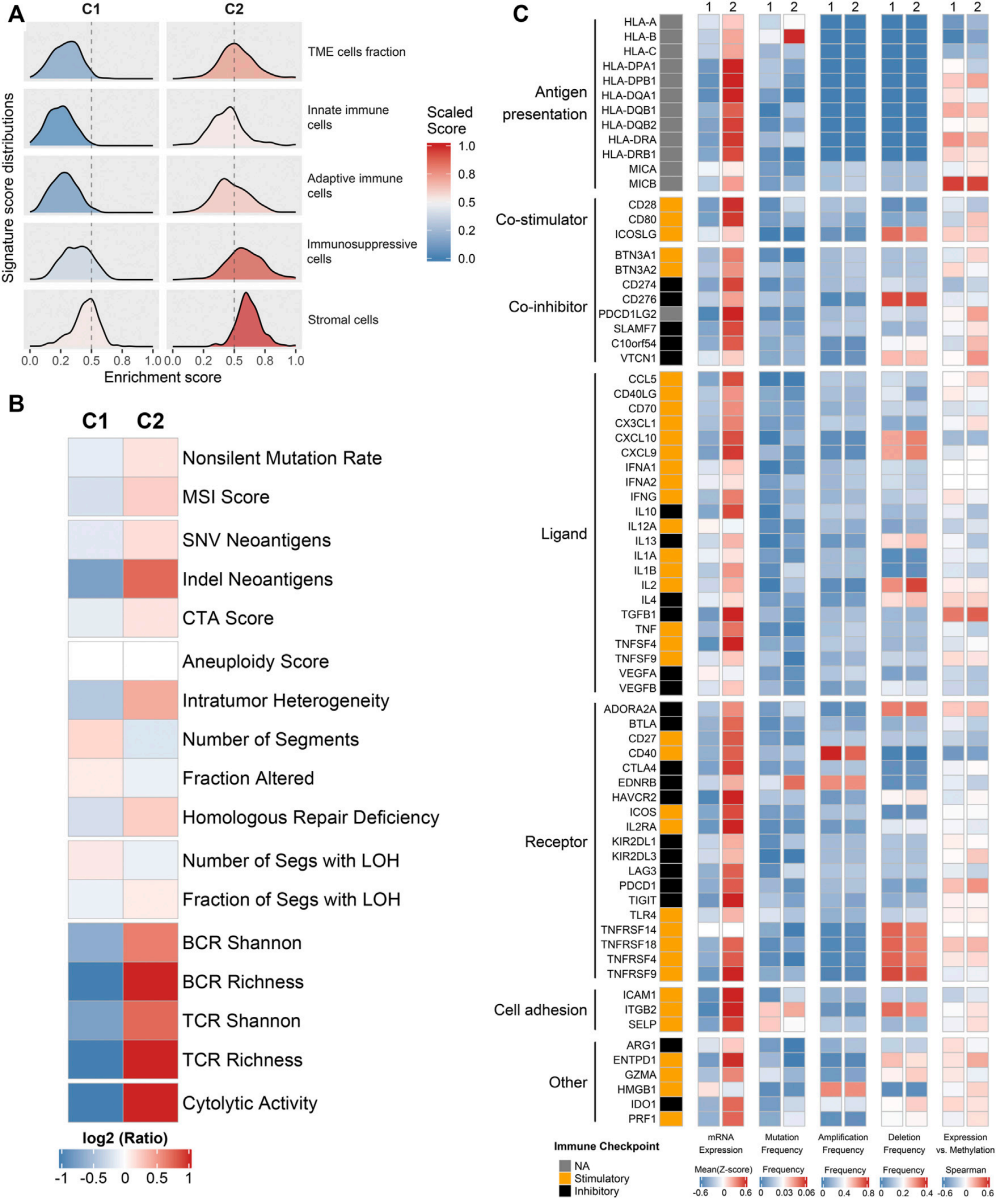
Potential immune escape mechanisms of each phenotype. **(A)** The relative abundance distributions of two immune subtypes in TME cells fraction, innate immune cells, adaptive immune cells, and stromal cells. **(B)** The expression levels of 17 tumor immunogenicity indicators of in C1 and C2. **(C)** Multi-omics analysis of 75 immunomodulators in two subtypes.

To further explore the regulatory mechanisms of the immune subtypes, we focused on the TCGA-CRC cohort, which possessed comprehensive omics data. We firstly investigated the exogenous immune escape mechanisms. Previous studies indicated that exogenous immune escape may include three major aspects: absence of leukocytes, presence of immunosuppressive cells, and release of abundant immunosuppressive cytokines (Schreiber et al., 2011; Beatty and Gladney, 2015). The relative abundance distributions of two immune subtypes in TME cells fraction, innate immune cells, adaptive immune cells and stromal cells were summarized in Figure 2A. C2 was characterized by higher levels of TME cell fraction, innate immune cells, adaptive immune cells and stromal cells. We also used leukocyte fraction and stromal fraction as indicators for further verification (Thorsson et al., 2018). The results are consistent with above, compared with C2, C1 showed lower levels in leukocyte fraction and stromal fraction (Supplementary Figure S5A,B). Therefore, it was speculated that the exogenous immune escape mechanism of C1 was ascribe to the lack of immune cells, while the exogenous immune escape mechanism of C2 was ascribe to the larger proportion of immunosuppressive cells and stromal cells.

### Intrinsic Immune Escape Mechanisms

We further explored the potential intrinsic immune escape mechanisms in two major facets: tumor immunogenicity and immune checkpoint molecules. The main elements of tumor immunogenicity are genome instability and antigen presentation ability. 17 elements associated with tumor immunogenicity were estimated. The heatmap and box plots illustrated the levels of these 17 indicators between C1 and C2 (Figure 2B and Supplementary Figures S5C–S **)**. Overall, C2 displayed higher immunogenicity relative to C1, such as BCR, TCR, cytolytic activity, and SNV and indel neoantigens (Figure 2B and Supplementary Figures S5C–S). To systematically measure the efficiency of antigen processing and presentation, we used the expression of MHC molecules (Figure 2C) and APS (Supplementary Figure S5T) as the main basis for evaluation. The results showed that C2 had higher APS and MHC-related molecules expression level compared with C1. Thus, the endogenous immune escape mechanism of C1 might be the low immunogenicity and impaired antigen presentation ability.

Furthermore, we further explored the expressions and multi-omics regulations of immunomodulators between two subtypes (Figure 2C). The results showed that C2 had both higher costimulatory and coinhibitory molecules than C1, which suggested C2 may upregulate the immune checkpoint molecules (such as CD274 and PDCD1LG2) to avoid immune surveillance. The mutation frequency of some indicators varies significantly between two phenotypes. For instance, HLA-B and EDNRB had a higher mutation frequency in C2 (Figure 2C). It was noteworthy that the differential expression of immunomodulators between the two subtypes could not be explained by CNV (all *p* > 0.05) (Figure 2C). The negative correlation between DNA methylation and gene expression indicated epigenetic silencing, such as CD80 (Figure 2C). The different characteristics of immunomodulators between immune subtypes provided clues for the discovery of new immunity therapy targets.

### Genomic Alterations of Immune Subtypes

**FIGURE 3:**
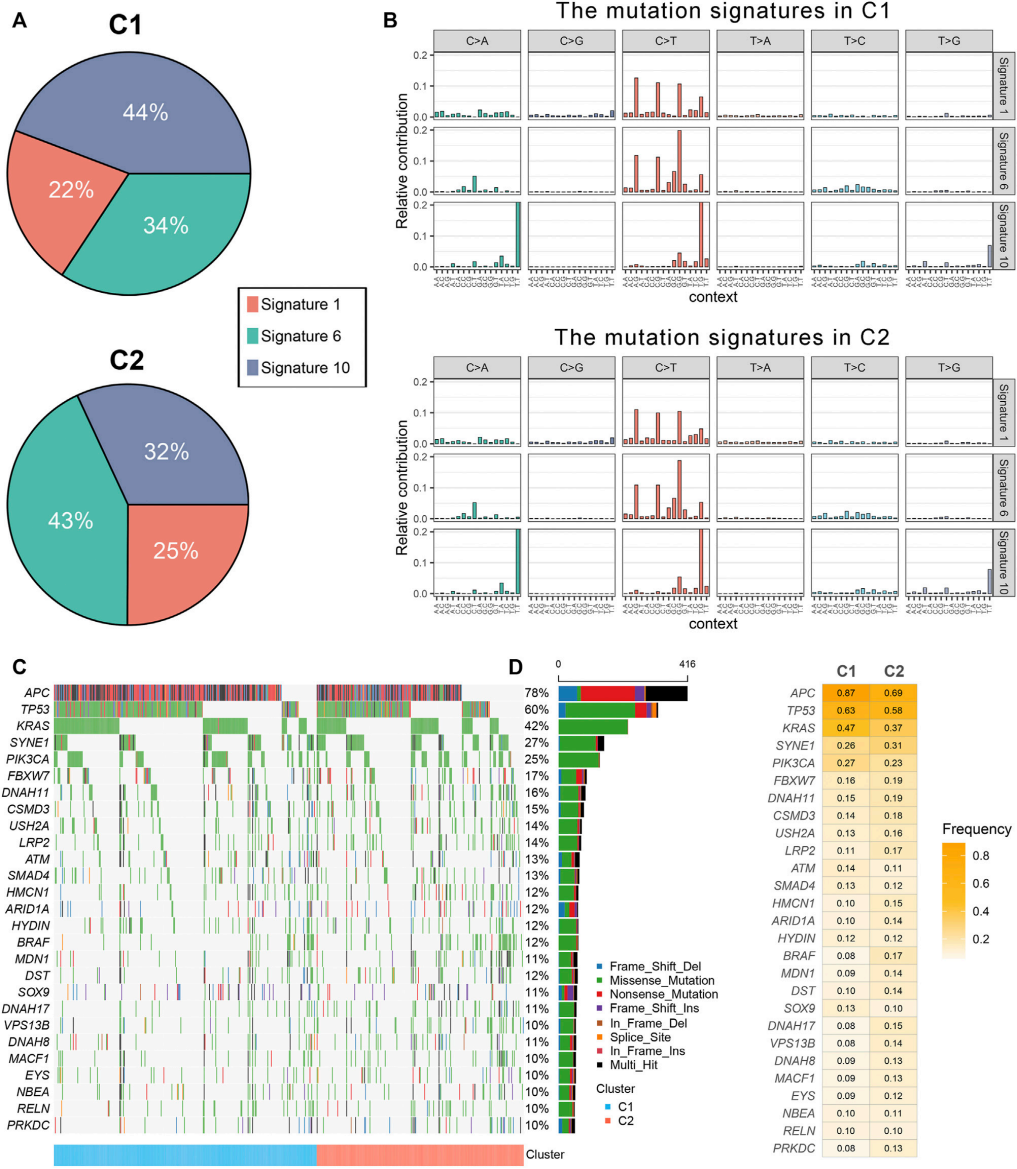
The mutational landscape of two immune subtypes. **(A)** Three mutation signatures were extracted from two immune subtype and named according to the COSMIC signature. The proportion of each mutation signature, which reflects the likely carcinogenic factors. **(B)** The relative contribution of three signature in C1 and C2. **(C,D)** The mutational landscape **(C)** and frequency **(D)** of 27 significant mutation genes (SMGs) in two subtypes.

**FIGURE 4:**
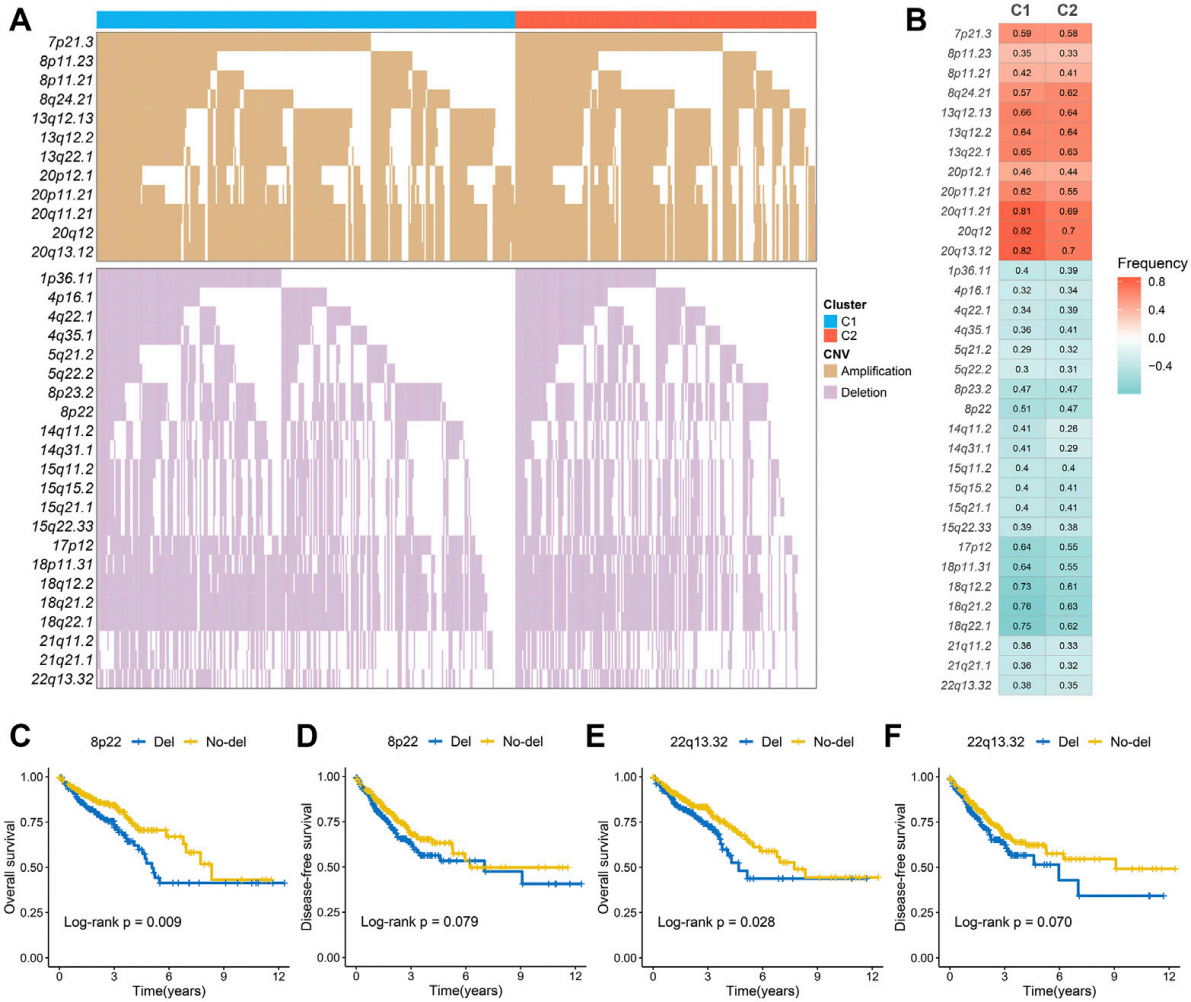
The copy number variations of two immune subtypes. **(A,B)** The waterfall plot **(A)** and alteration frequency **(B)** of significantly amplified and loss chromosomal segments in C1 and C2. **(C,D)**. Kaplan-Meier survival analysis of overall survival **(C)** and disease-free survival **(D)** according to the 8p22 deletion. **(E,F)** Kaplan-Meier survival analysis of overall survival **(E)** and disease-free survival **(F)** according to the 22q13.32 deletion.

The mutation spectrums were decoded to analyze its potential biological carcinogenic factors. The NMF package was used to identify three mutation signatures for two immune subtypes (Figure 3A). The cosine similarity between the extracted mutation signature and thirty COSMIC signatures were shown in Supplementary Figure S6A-B (Alexandrov et al., 2015). Figure 3B showed the proportion of each mutation signature, which reflects the predominant carcinogenic factors. Signature 10 accounted for the highest proportion in C1, indicating that carcinogenic factor was mainly related to altered activity of the error-prone polymerase POLE. Signature six accounted for the highest proportion in C2, indicating that carcinogenic factor was mainly related to microsatellite instability (MSI). Additionally, although not significant, the tumor mutation burden (TMB) of C2 was greater than C1 (Supplementary Figure S6C). In total, 27 FMGs were identified (Figure 3C). The co-occurrence or elusive of these 27 genes were shown in Supplementary Figure S6D. Univariate cox regression further revealed the prognostic value of these 27 FMGs (Supplementary Figure S7A,B). Among these genes, USH2A and KRAS were poor prognostic factors. In addition, we also investigated the mutation frequency of FMGs in each cluster. It was found that mutations in APC, TP53, and KRAS were enriched in C1 although C2 had the higher TMB (Figure 3D). GISTIC 2.0 recognized the significantly amplified and loss chromosomal segments in the TCGA-CRC cohort (Supplementary Figures S8A,B). The results showed that there was no significant difference in the CNV load of the two immune subtypes (Supplementary Figure S8C). Of the 34 driver segments, 12 were amplified and 22 were loss (Figure 4A). C1 was characterized by the more frequent alterations encompassing 20p11.21, 20q11.21, 20q12, and 20q13.12 amplifications as well as 17p12, 18p11.31, 18q12.2, 18q21.2, and 18q22.1 loss (Figure 4B). Univariate Cox regression further revealed the prognostic value of these 34 segments (Supplementary Figures S9A,B). Kaplan-Meier survival analysis suggested that the deletions of 8p22 and 22q13.32 were significantly associated with poor OS and DFS (Figures 4C–F).

### Clinical Characteristics of Different Immune Subtypes

**FIGURE 5:**
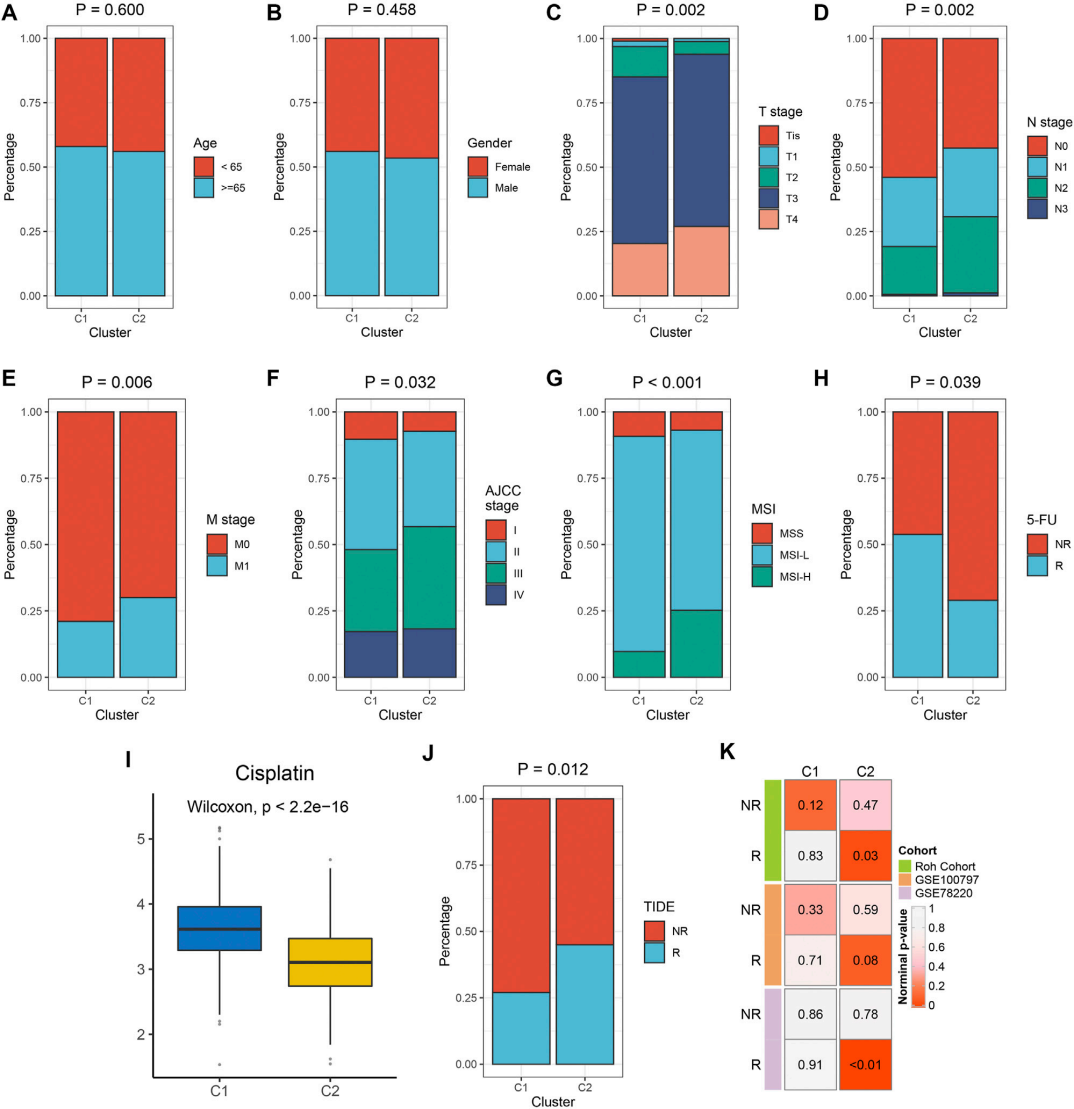
The clinical significance of the two immune subtypes in the *meta*-GEO cohort. **(A–H)** Composition percentages of clinical characteristics such as age **(A)**, gender **(B)**, T stage **(C)**, N stage **(D)**, M stage **(E)**, AJCC stage **(F)**, MSI **(G)**, and 5-FU response **(H)** between C1 and C2. **(I)** The IC50 distribution of Cisplatin between two subtypes. **(J)** Composition percentages of immunotherapy response estimated by TIDE algorithm between C1 and C2. **(K)** Submap analysis revealed that C2 was sensitive to immunotherapy.

We examined the distribution of clinical characteristics including age, gender, TNM stage, AJCC-stage, MSI, and 5-FU response rates. There was no significant difference in age and gender distribution among the two subtypes. C1 had a higher response rate of 5-FU. C2 had higher levels of T stage, N stage, distant transfer, AJCC-stage, and MSI-status (Figures 5A–H). The prediction of pRRophetic indicates that C2 was more sensitive to Cisplatin (Figure 5I). The previous results indicate that C2 belonged to the immune hot subtype but was in an immunosuppressive state; C1 belonged to the immune cold subtype. Therefore, we further explored the sensitivity of immune phenotypes to immunotherapy. The TIDE algorithm showed that C2 had a higher proportion of responders to immunotherapy (Figure 5J). SubMap also showed that C2 was more prone to respond to immunotherapy (Figure 5K). Supplementary Figure S10 showed similar results in the TCGA-CRC validation set.

### Development of Prognosis Associated Risk Score

**FIGURE 6:**
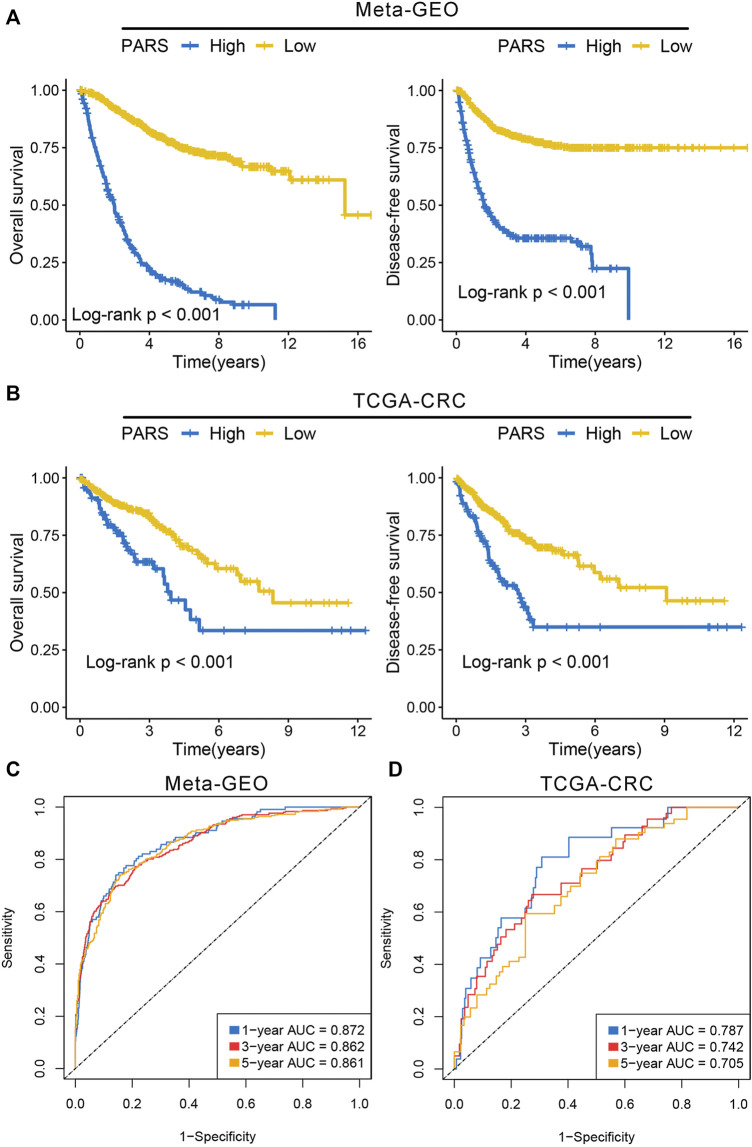
Development of prognosis associated risk score. **(A)** Kaplan-Meier survival analysis of overall survival and disease-free survival according to PARS in the *meta*-GEO cohort. **(B)** Kaplan-Meier survival analysis of overall survival and disease-free survival according to PARS in the TCGA-CRC cohort. **(C,D)** ROC curves of PARS in the *meta*-GEO **(C)** and TCGA-CRC **(D)** cohorts.

We identified 388 and 572 DEGs in *meta*-GEO cohorts and TCGA-CRC cohort, respectively (Supplementary Figure S11A,B). The overlapping DEGs in both cohorts eventually determined 312 CDEGs (Supplementary Figure S11C). The biological process (BP) and KEGG pathway enrichment analysis of these CDEGs revealed plenty of immune related functions such as cytokine-cytokine receptor interaction, response to stimulus and immune system process (Supplementary Figures S11D,E). Based on the constructed pipeline, we further transformed the CDEGs expression matrix into the gene pairs matrix, and further screened 980 gene pairs with significantly prognosis significance (adjust-*p* <0.05; Supplementary Table S6). Subsequently, the Lasso regression was performed to develop the optimal model, and it was determined by the optimal lambda = 0.0324 (Supplementary Figure S11F and Supplementary Table S7). We calculated the PARS of each patient, and divided the patients into high risk and low risk groups. The Kaplan-Meier analysis suggested the patients with high PARS tended to possess a worse OS and DFS relative to patients with low PARS in both *meta*-GEO cohorts and TCGA-CRC cohort (Figures 6A,B). The area under the ROC curves (AUCs) of predicting 1-year, 3 years, and 5 years OS were 0.872, 0.862 and 0.861 in the *meta*-GEO cohort, 0.787, 0.742 and 0.705 in the TCGA-CRC cohort (Figures 6C,D). The C-index was 0.815 [95%CI: 0.795–0.835] and 0.738 [95%CI: 0.675–0.801] in the *meta*-GEO cohort and TCGA-CRC cohort, respectively. We also found C1 had lower PARS compared with C2 in both meta cohorts and TCGA cohort (Supplementary Figures S11G,H), which was in line with the prognostic characteristic of immune subtypes. These results indicated PARS was a robust and promising signature for prognosis.

### Construction of a Nomogram for Evaluating Prognosis

**FIGURE 7:**
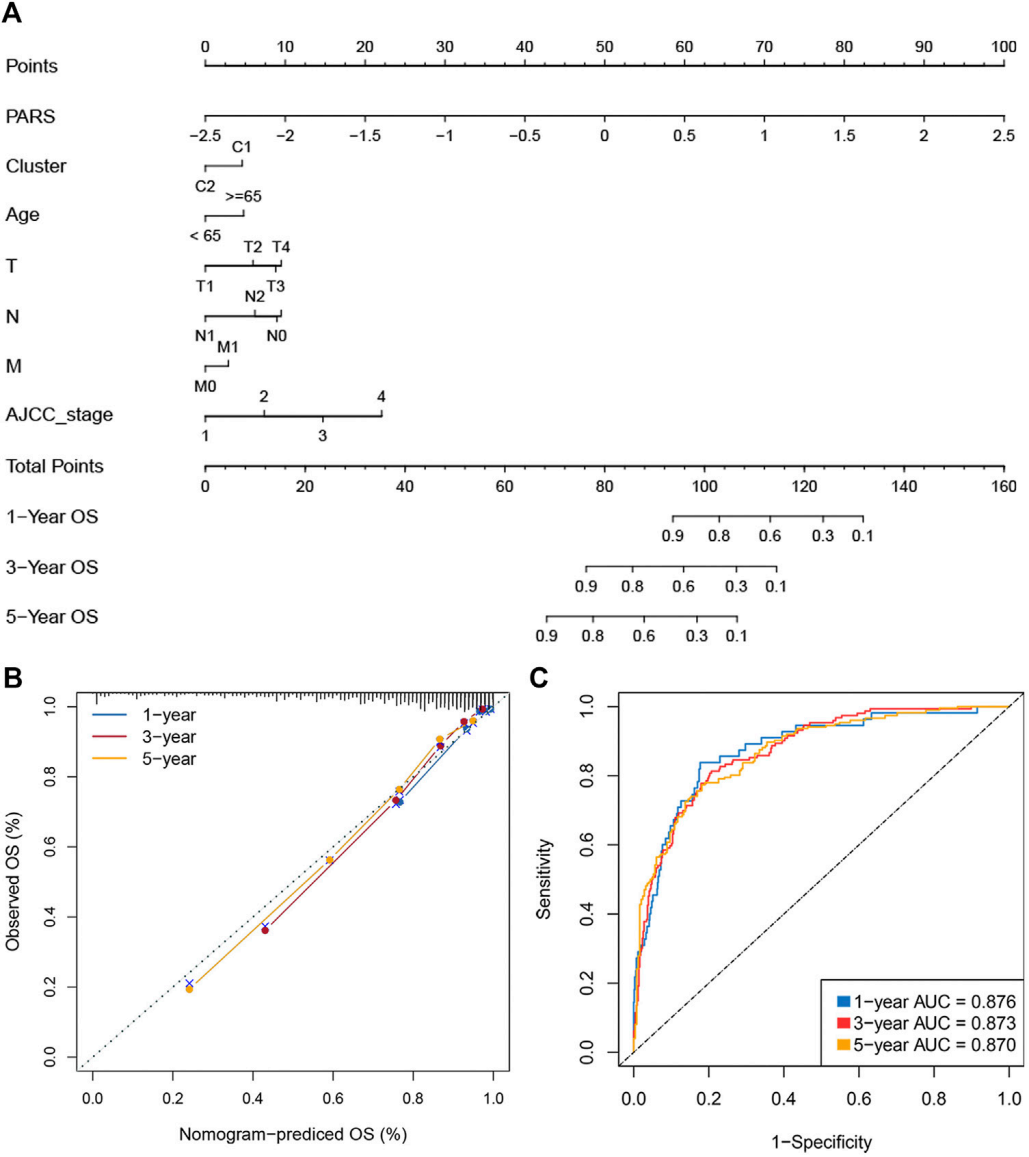
Construction of a nomogram for evaluating prognosis. **(A)** Nomogram for predicting the 1-, 3-, and 5 years OS of CRC patients. **(B)** Calibration analysis of our nomogram in evaluating the 1-, 3-, and 5years OS. **(C)**. ROC curve for evaluating the performance of nomogram in predicting the 1-, 3-, and 5 years OS.

The immune subtypes, age, gender, TNM stage, AJCC stage, and MSI status were subjected into the univariate Cox regression analysis of OS and DFS. Multivariate cox regression analysis found that PARS and Cluster are independent prognostic factors of OS. For DFS, only PARS was an independent prognostic factor (Supplementary Table S8). We selected statistically significant variables to further construct nomograms (Supplementary Table S8 and Figures 7A,B). The nomogram was used to assess the 1-, 3-, and 5 years survival rates of a single patient. We use ROC and calibration plots to evaluate the nomogram. The calibration curves showed a good assistant between the nomogram prediction and the observed value (Figure 7B). The AUCs for 1-, three- and 5-years were 0.876, 0.873, and 0.870, respectively (Figure 7C). These results indicated that the nomograms had excellent performance. The above indicated the nomogram was reliable, which could facilitate the clinical managements of CRC.

## Discussion

More and more patients with solid tumors benefit from immunotherapy (Doi et al., 2018; Song et al., 2018; Ganesh et al., 2019). However, the effective response and survival benefits to immunotherapy are usually limited to a small subset of patients. In this study, we identified two robust CRC immune subtypes through consensus clustering and found that each immune subtype had distinct immune escape mechanisms, genome alterations, and clinical characteristics. This study provides an innovative CRC classification concept, and immunological classification may have clinical guiding significance for personalized immunotherapy. Our work reflects innovation in several important ways.

Firstly, the unsatisfactory response efficiency of immunotherapy might be due to tumor immune escape. Therefore, it was very necessary to explore the immune escape mechanisms of different immune subtypes. We analyzed the cellular and molecular characteristics of these two immune subtypes. It was found that the exogenous immune escape mechanism of C1 was the lack of immune cells, especially immune killer cells, while the exogenous immune escape mechanism of C2 was the increase of immunosuppressive cells and stromal cells. In addition, the endogenous immune escape of C1 was mainly due to low immunogenicity and impaired antigen presentation ability. C2 was more immunogenic, but the increase of immunosuppressive molecules may be the reason for its endogenous immune escape. Different immune escape mechanisms might be the key impediments to the development of immunotherapy for two subtypes.

Secondly, different carcinogenic factors lead to different mutation spectrums. Therefore, we tried to analyze its potential biological carcinogenic factors through the tumor mutation spectrum. We found that signature 10 has the highest proportion in C1, indicating that carcinogens are mainly related to changes in the activity of the error-prone polymerase POLE. Signature six accounted for the highest proportion in C2, indicating that its main carcinogenesis was related to MSI. By identifying FMGs, we found APC, TP53, and KRAS mutations were enriched in C1 although C2 had the higher TMB. In addition, C1 was characterized by the more frequent alterations encompassing 20p11.21, 20q11.21, 20q12, and 20q13.12 amplifications as well as 17p12, 18p11.31, 18q12.2, 18q21.2, and 18q22.1 loss. These results revealed the molecular landscape of two subtypes.

Next, our research provided clues for choosing clinical treatment options. Analysis of clinical characteristics of different immune subtypes may help to accurately select chemotherapy drugs. C1 has a higher response rate of 5-FU while C2 was more sensitive to Cisplatin. In addition, our results might facilitate the selection of suitable patients for immunotherapy. Two algorithms including TIDE and SubMap demonstrated that C2 was more likely to respond to immunotherapy.

Finally, we proposed a gene pair pipeline to develop a predictive model. The gene pair was concerned about the mathematical relationship between the mRNA expression of two genes and ignored the batch effects of different platforms and facilitated the clinical application. Our PARS model had the accurate performance for predicting OS. To further advance the managements of CRC, we constructed a nomogram for evaluating individual patient risk. Overall, our PARS and nomogram displayed stable and robust performance in the *meta*-GEO and TCGA-CRC cohorts and might be promising tools in clinical settings.

Prior to this study, a few reports identified molecular subtypes based on gene expression profiles or mutational signatures (Guinney et al., 2015; Dunne et al., 2017; Liu et al., 2021h). To the best of our knowledge, this is the first study to date comprehensively delineating the immune and molecular landscape of CRC according to the expression files of scale sample and the broad-spectrum immune genes. Two identified immune subtypes displayed substantial differences in immunology, genomic alterations, and clinical features. This raises the intriguing issue of how to optimally regulate the host immune response so that patients are mobilized toward more favorable states, providing a roadmap to more successful immunotherapy. Combined with the difference in Cisplatin sensitivity and molecular alterations between the two subtypes, this may provide references for precise treatment of CRC. Thus, this study has potential therapeutic implications for the rational design of combination immunotherapy strategies. Although our cluster is promising, some limitations should be acknowledged. First, due to lack of data, we only considered the inter-individual heterogeneity, but did not consider the intra-tumor heterogeneity. Second, the sensitivity to Cisplatin and immunotherapy was evaluated via machine learning algorithms, further clinical validation is necessary.

In conclusion, our research provides a new classification strategy for CRC. The two subtypes were characterized by distinct immune escape mechanisms, molecular alterations, clinical characteristics, and prognosis. Additionally, our PARS and nomogram were robust and promising indicators for assessing the prognosis of CRC patients. Our study provided deep insights and novel clinical management strategies for CRC.

## Data Availability

The datasets presented in this study can be found in online repositories. The names of the repository/repositories and accession number(s) can be found in the article/Supplementary Material.

## Abbreviations

CRC: Colorectal cancer; GEO: Gene Expression Omnibus; TCGA: The Cancer Genome Atlas; ssGSEA: Single sample gene set enrichment analysis; CDF: Cumulative distribution function; PAC: Proportion of ambiguous clustering; IGP: In-group proportion; GSVA: Gene set variation analysis; TMB: Tumor mutation burden; AS: Aneuploidy scores; HRD: Homologous recombination defects; MSI: Microsatellite instability; CTAs: Cancer/testis-antigens; APS: Antigen processing and presenting machinery scores; CNV: Copy number variation; SMGs: Significant mutation genes; NMF: Non-negative matrix factorization; DEGs: Differentially expressed genes; OS: Overall survival; DFS: disease free survival; ROCs: Receiver operating characteristic curves; AUC: Area under the ROC curve.
