# Supplementary material for: Immune Landscape Refines the Classification of Colorectal Cancer With Heterogeneous Prognosis, Tumor Microenvironment and Distinct Sensitivity to Frontline Therapies
Source: Front Cell Dev Biol. 2022 Jan 10;9:784199. doi: 10.3389/fcell.2021.784199 (PMC8784608; doi:10.3389/fcell.2021.784199)
Supplement: Supplementary file 1 [file DataSheet1.docx]

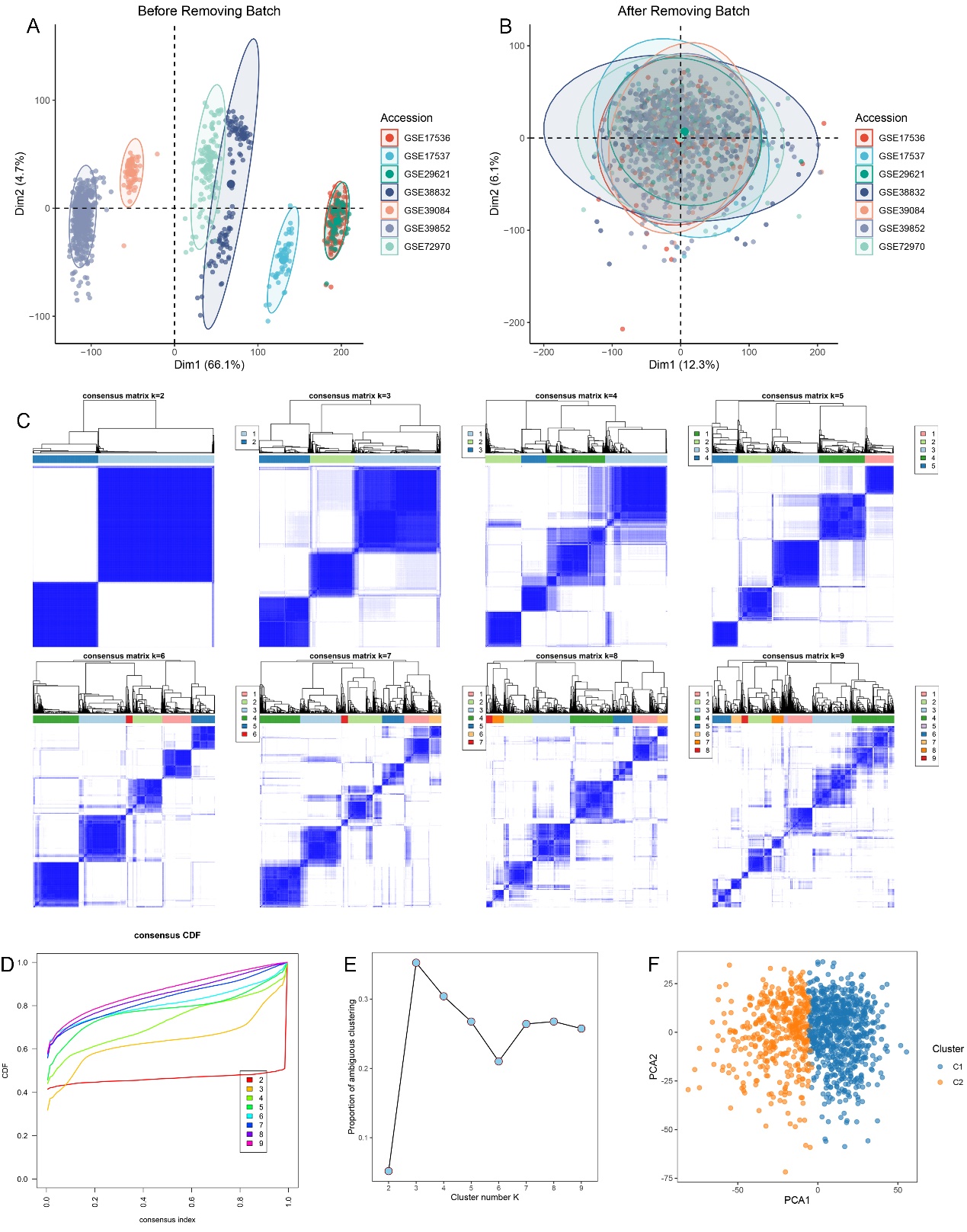


**Supplementary Figure 1 |** Identification of two immune subtypes. **A-B**. The spatial distribution of seven GEO datasets before (**A**) and after (**B**) removing batch effects. **C.** The consensus clustering of CRC samples based on immune-related gene expression profiles in the meta-GEO datasets. **D-E.** The CDF curves (**D**) and PAC (**E**) analysis to identify robust immune subtypes. **F.** The two-dimensional PCA analysis of two immune phenotypes.


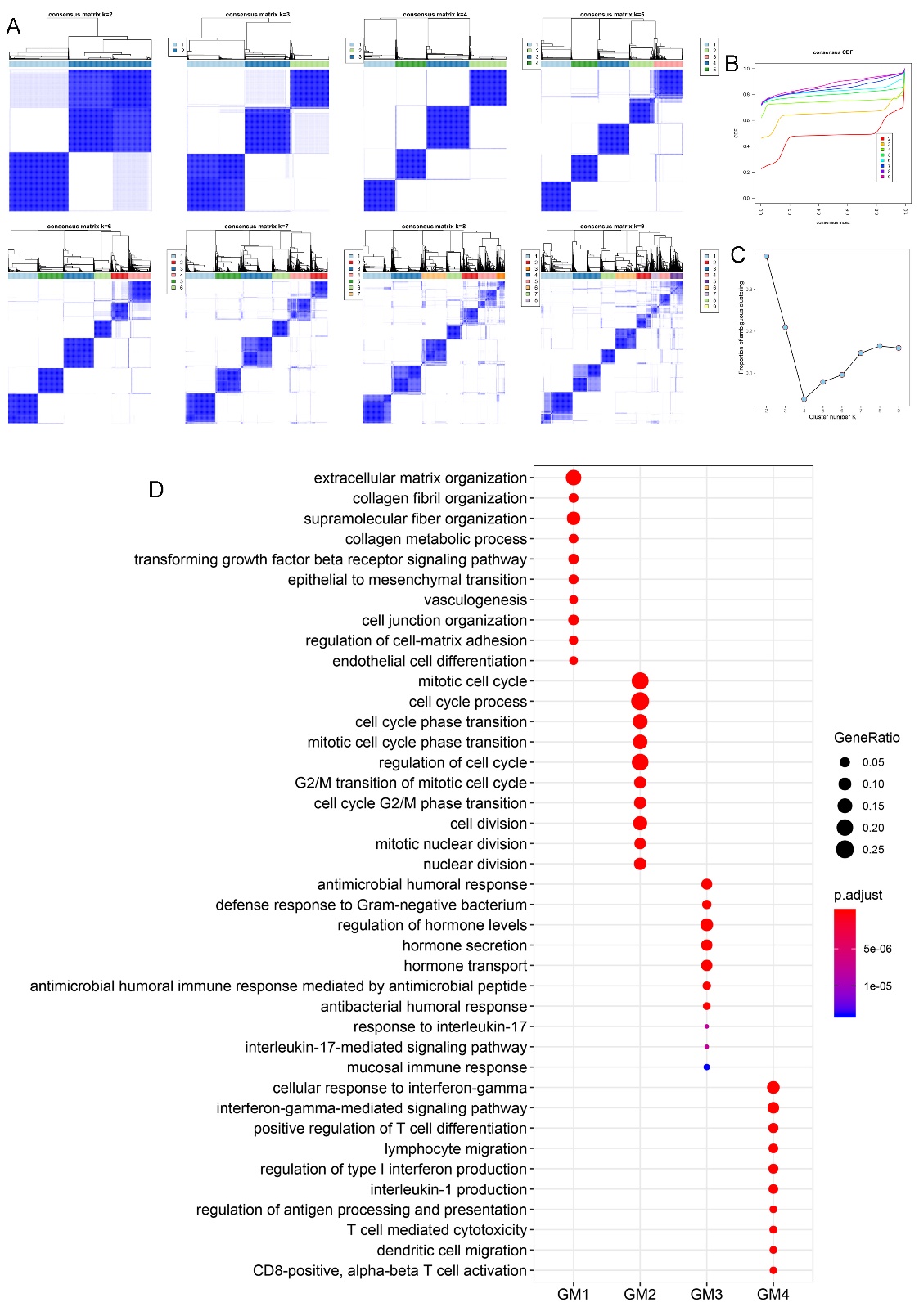


**Supplementary Figure 2 | Identification of four gene modules. A.** The consensus clustering was applied to identify gene modules. **B-C.** The CDF curves (**B**) and PAC (**C**) analysis to identify gene modules. **D.** GO enrichment analysis was performed on these four gene modules.


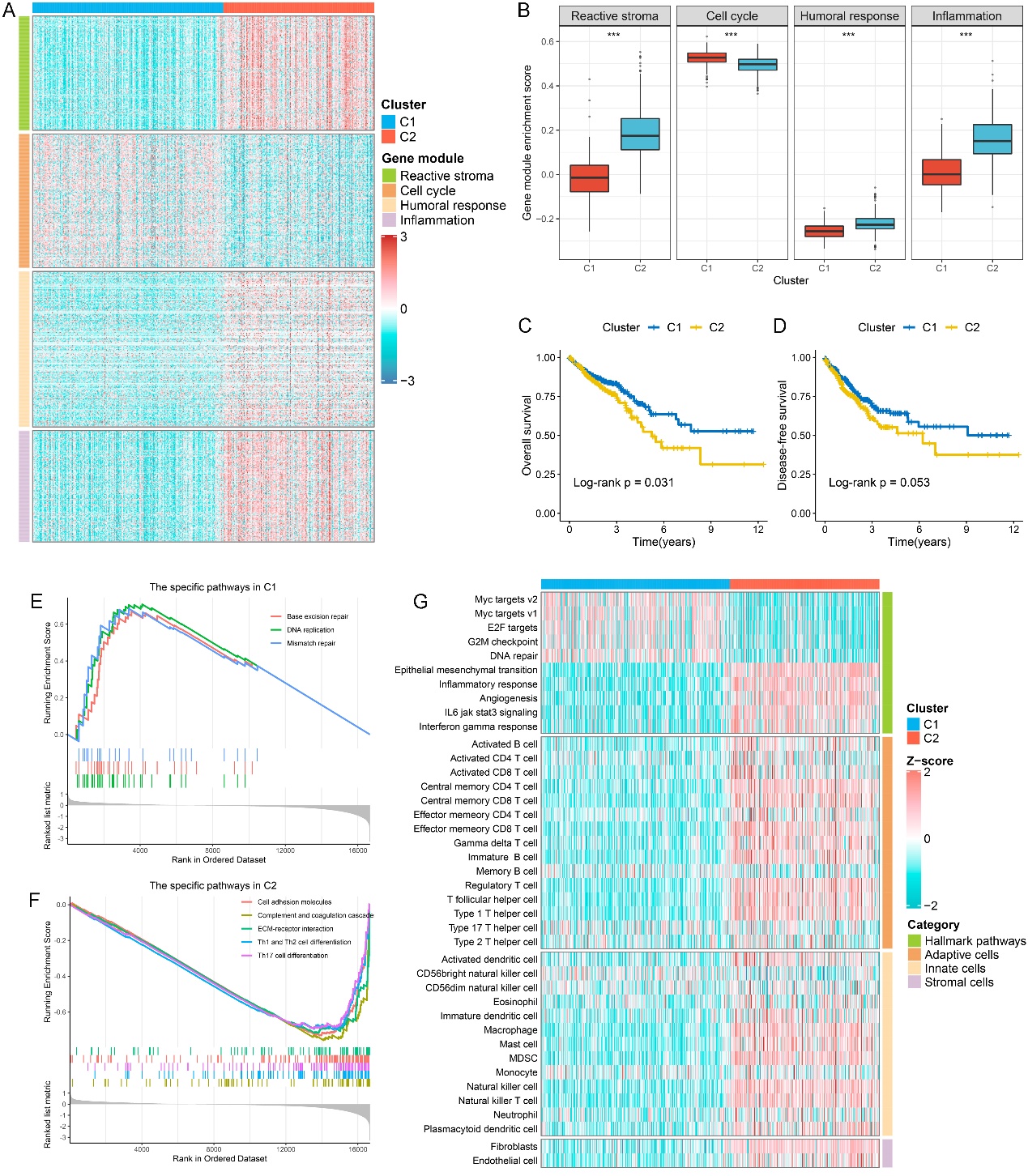


**Supplementary Figure 3 |** The specific functions and survival status of each subtype in the TCGA-CRC cohort. **A**. The expression profiles of gene modules between two subtypes. **B**. The ssGSEA algorithm was performed to quantify the relative abundance of four gene modules between two subtypes. **C-D**. Kaplan-Meier survival analysis of overall survival (**C**) and disease-free survival (**D**) according to the two subtypes. **E-F**. GSEA was performed to identify specific KEGG pathways in C1 (**E**) and C2 (**F**). **G.** The hallmark analysis (GSVA) and immune cell infiltration estimation (ssGSEA) of two subtypes.


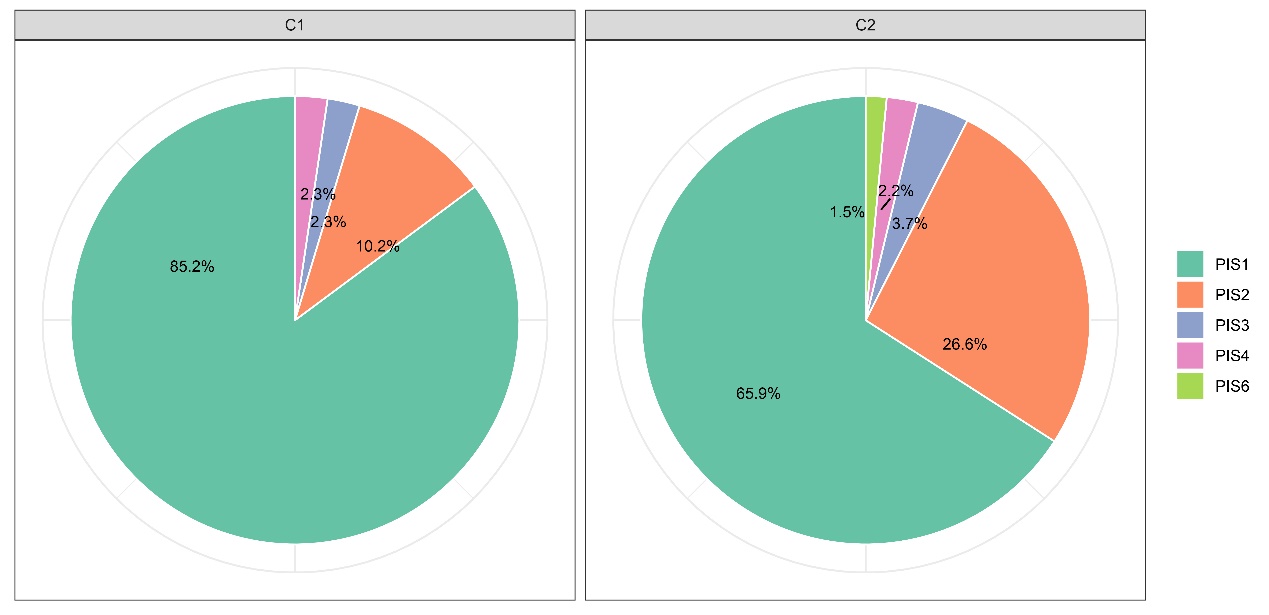


**Supplementary Figure 4 |** The distribution of pancancer immune landscape subtypes in our subtypes.


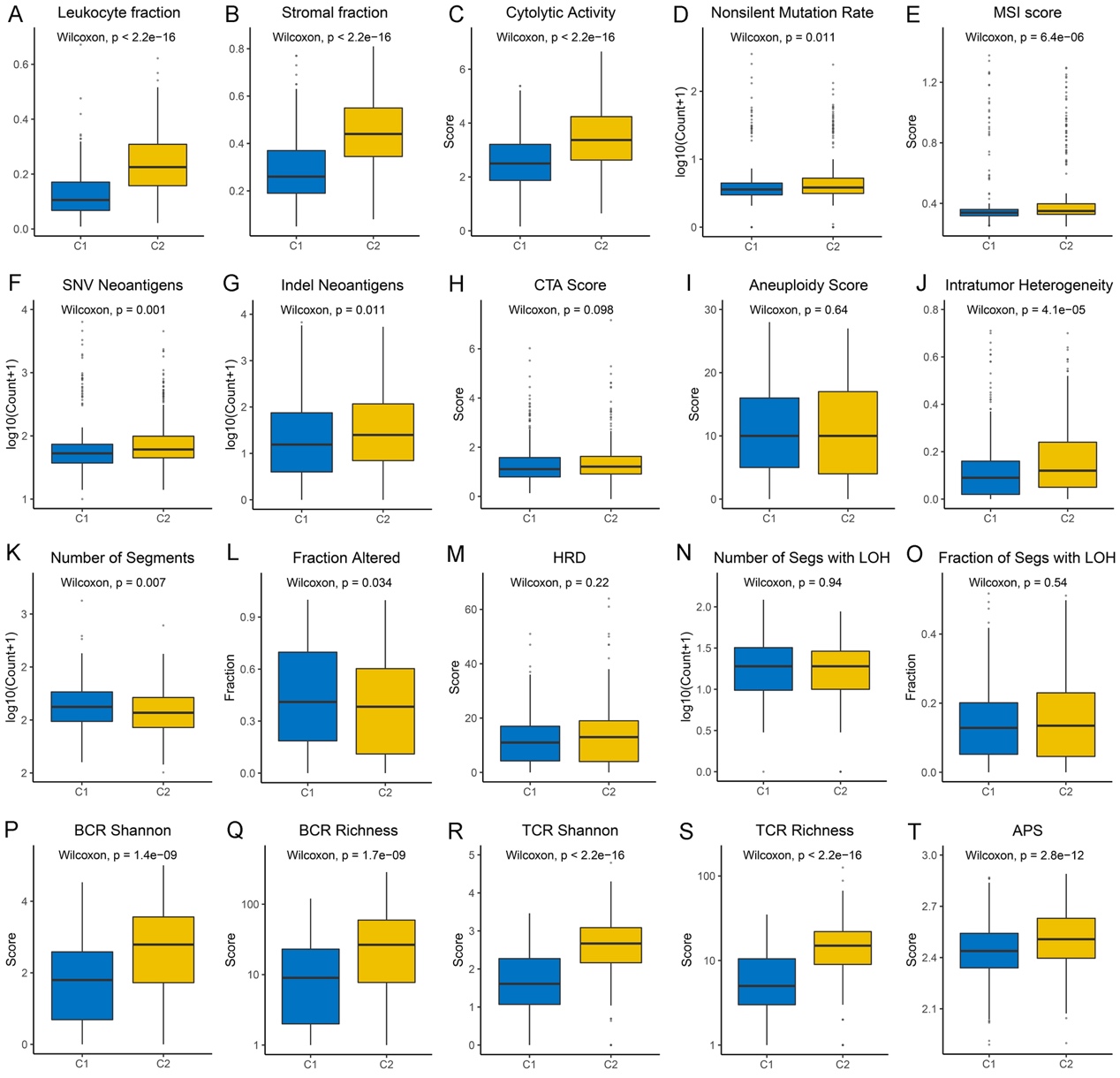


**Supplementary Figure 5 |** Potential immune escape mechanisms of each phenotype. The relative abundance distributions of two immune subtypes in leukocyte fraction (**A**), stromal fraction (**B**), cytolytic activity (**C**), nonsilent mutation rate (**D**), MSI score (**E**), SNV neoantigens (**F**), indel neoantigens (**G**), CTA score (**H**), aneuploidy score (**I**), intratumor heterogeneity (**J**), number of segments (**K**), fraction altered (**L**), HRD (**M**), number of segments with LOH (**N**), fraction of segments with LOH (**O**), BCR Shannon (**P**), BCR Richness (**Q**), TCR Shannon (**R**), TCR Richness (**S**), and APS (**T**).


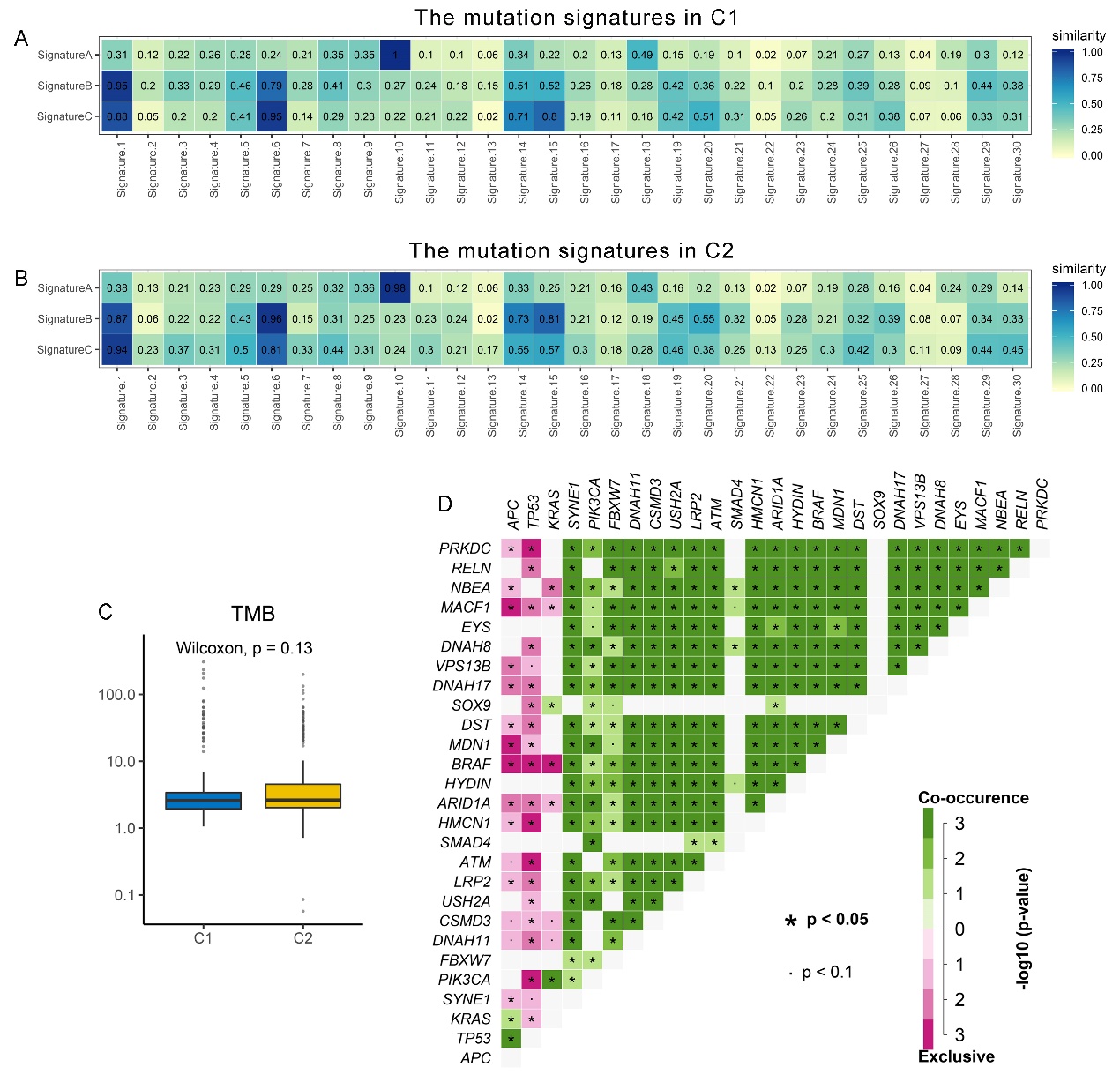
 **Supplementary Figure 6 |** The mutation signatures in C1 and C2. **A-B.** The cosine similarity between the extracted mutation signature and thirty COSMIC signatures in C1 (**A**) and C2 (**B**). **C.** Distribution of tumor mutation burden (TMB) between C1 and C2. **D.** The co-occurrence or elusive of 27 FMGs.


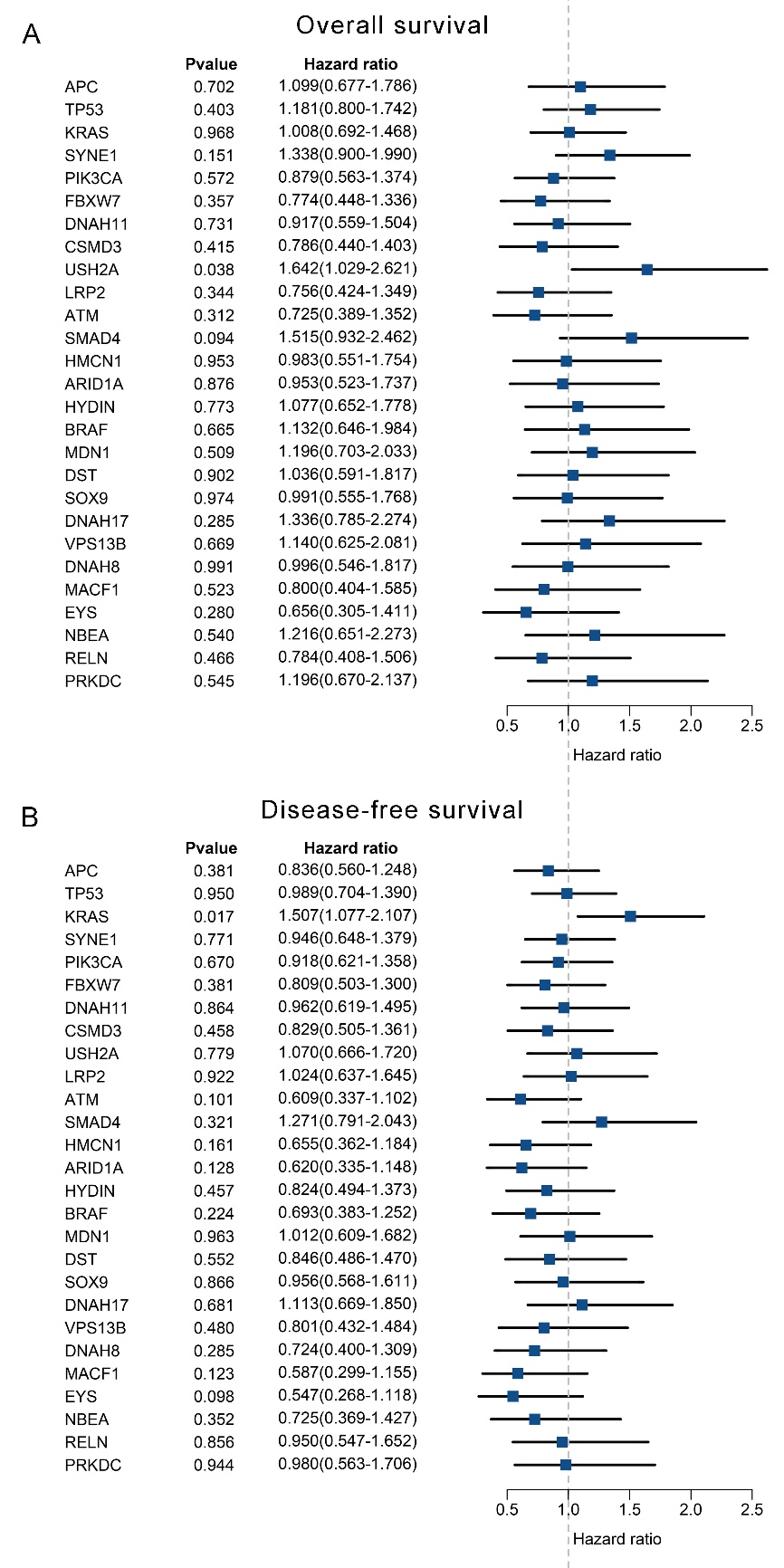


**Supplementary Figure 7 |** Univariate cox regression and survival analysis of 27 driver FMGs for overall survival (**A**) and disease-free survival (**B**).


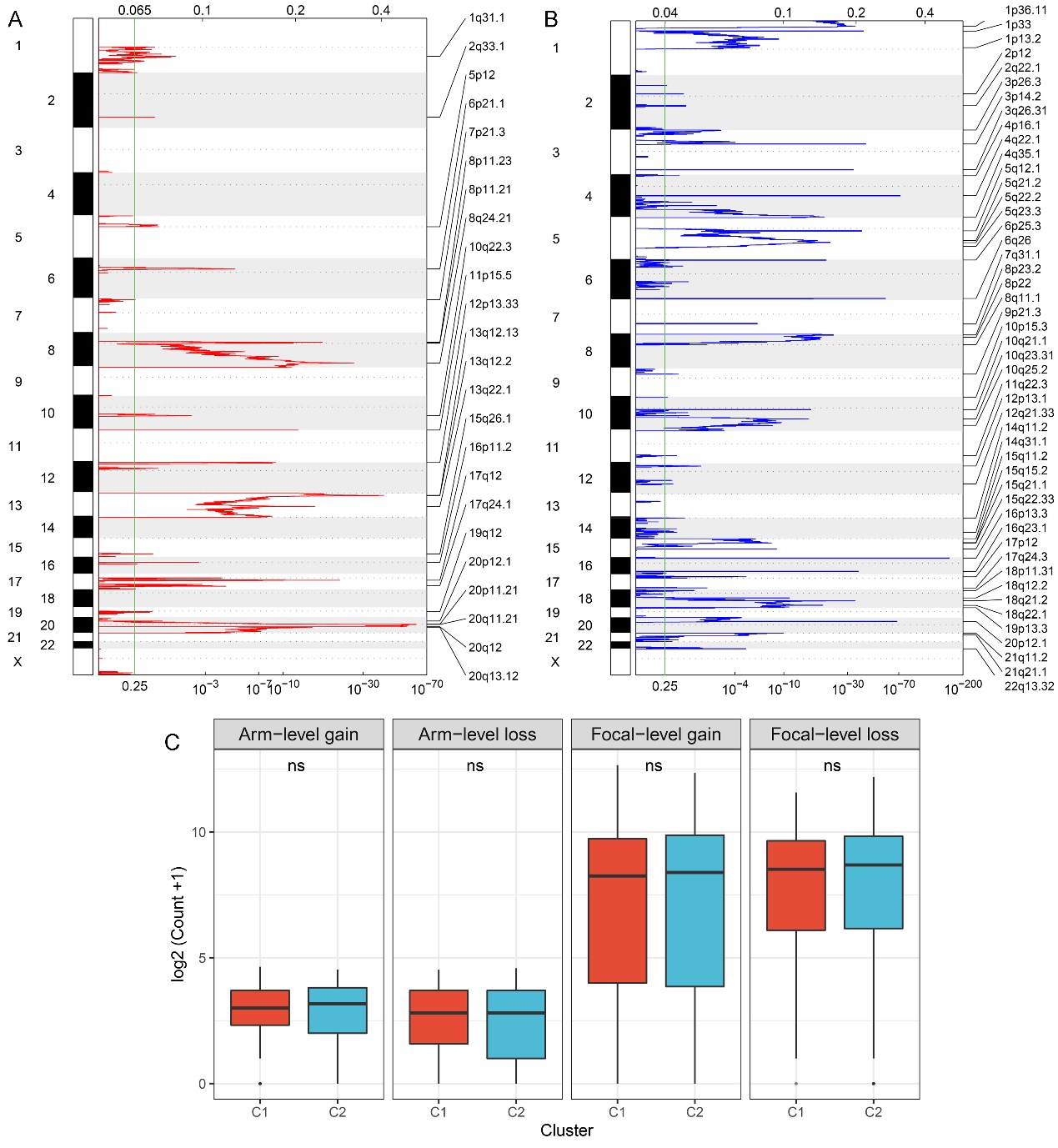


**Supplementary Figure 8 |** The copy number variations of two immune subtypes. **A-B.** The significantly amplified (**A**) and loss (**B**) chromosomal segments in TCGA-CRC. **C.** The copy number variation load of the two immune subtypes.


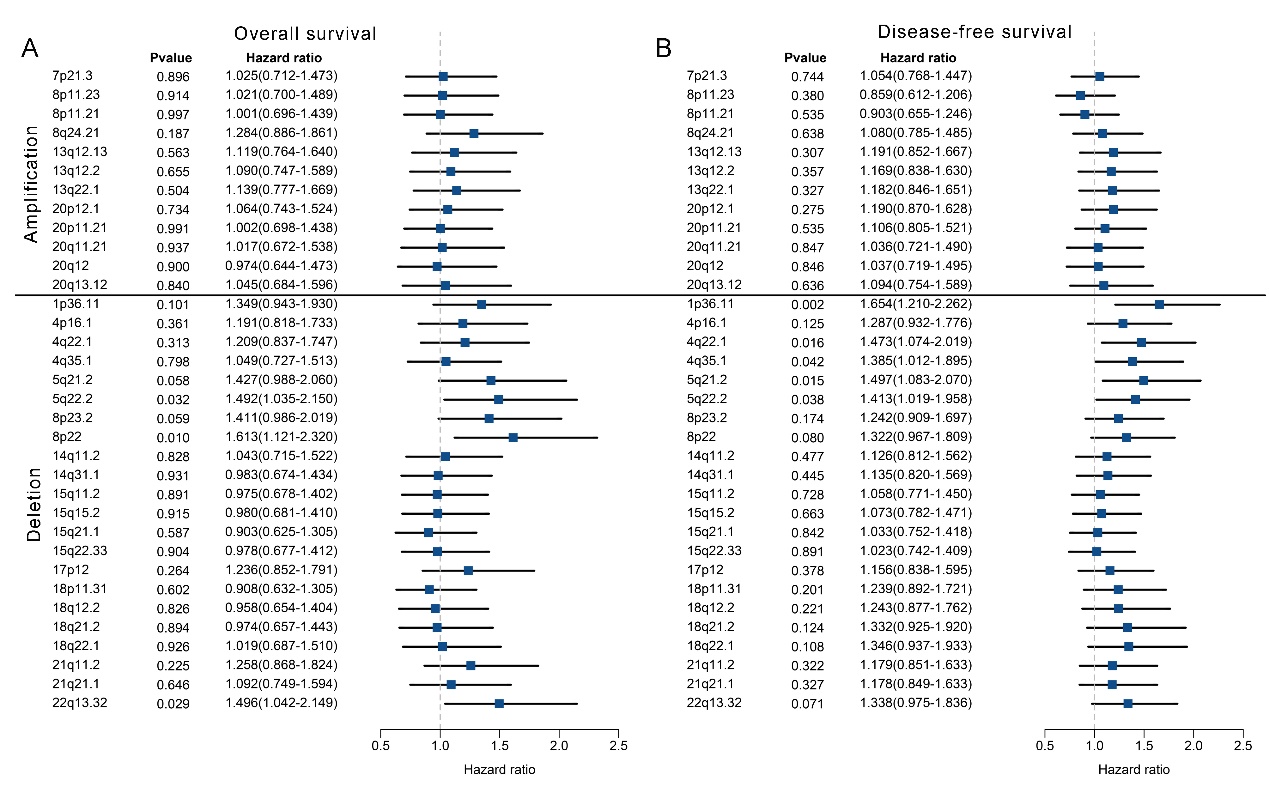


**Supplementary Figure 9|** Univariate cox regression and survival analysis of significantly altered segments for overall survival (**A**) and disease-free survival (**B**).


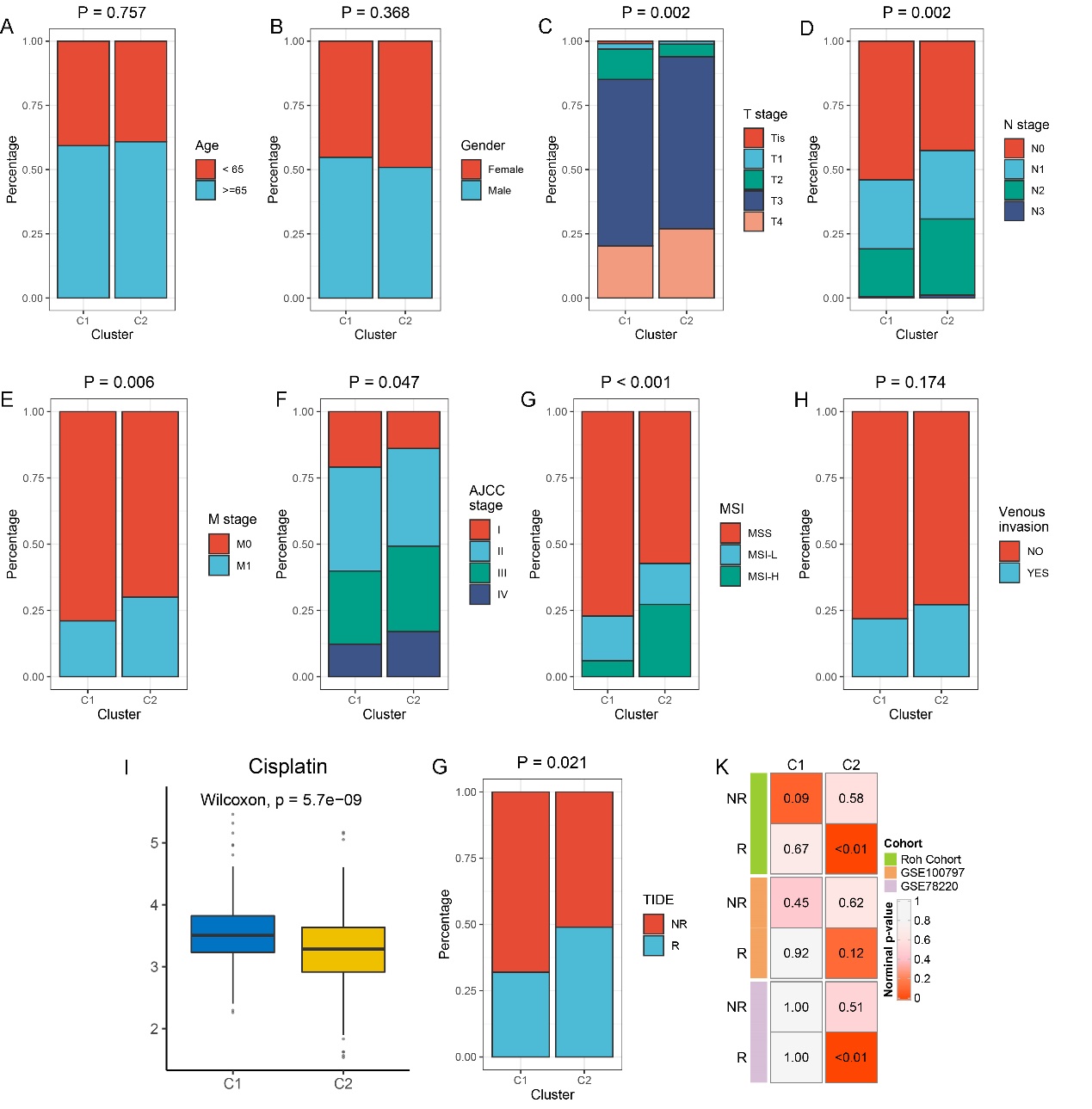


**Supplementary Figure 10** **|** The clinical significance of the two immune subtypes in the TCGA-CRC cohort. **A-H.** Composition percentages of clinical characteristics such as age (**A**), gender (**B**), T stage (**C**), N stage (**D**), M stage (**E**), AJCC stage (**F**), MSI (**G**), and venous invasion (**H**) between C1 and C2. **I**. The IC50 distribution of Cisplatin between two subtypes. **J.** Composition percentages of immunotherapy response estimated by TIDE algorithm between C1 and C2. **K.** Submap analysis revealed that C2 was sensitive to immunotherapy.


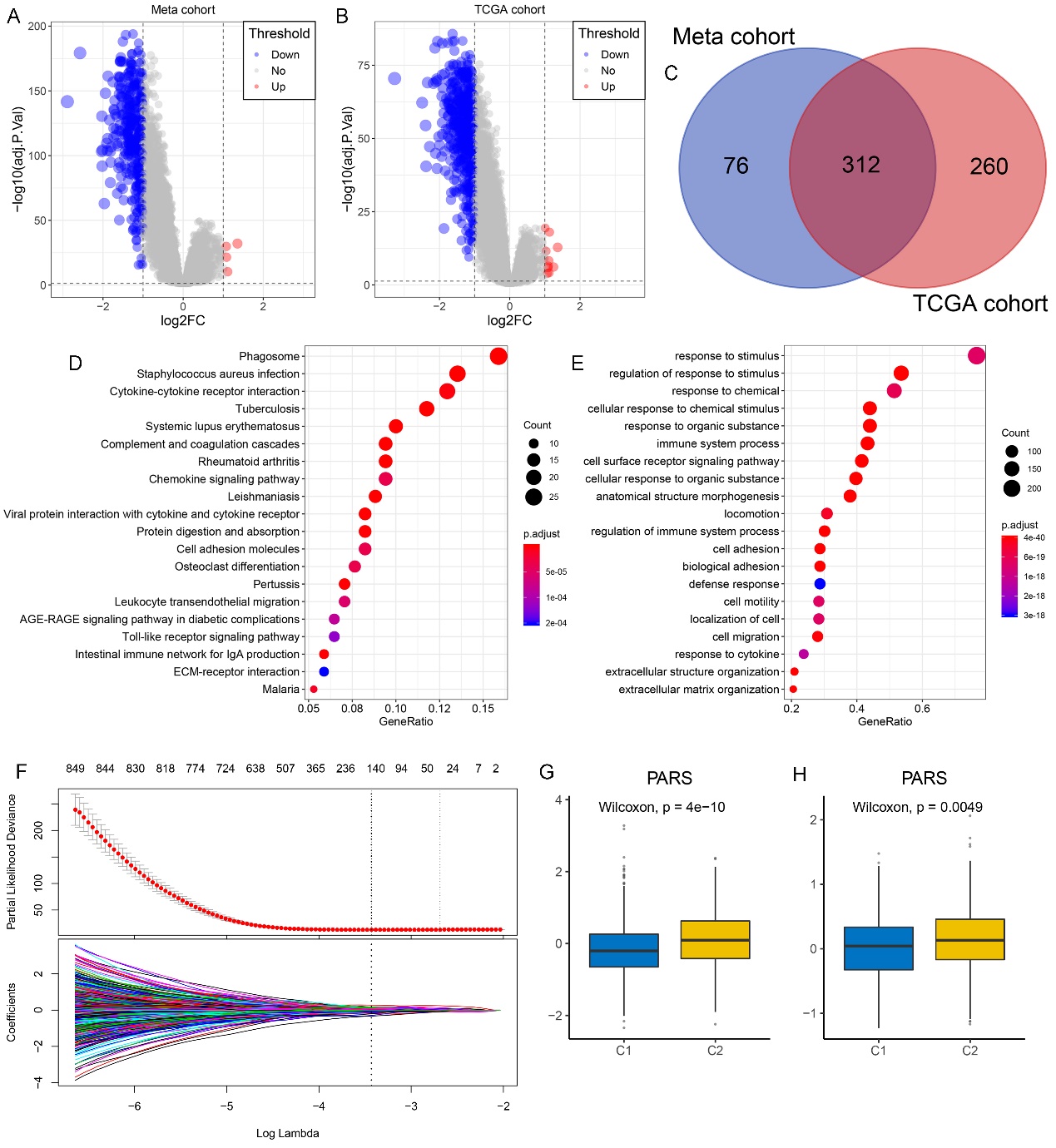
 **Supplementary Figure 11** **|** **Development of prognosis associated risk score.** A-B. Differential expression analysis between C1 and C2 in meta-GEO (**A**) and TCGA-CRC (**B**) cohorts. **C**. The overlapping DEGs in two cohorts. **D-E.** The biological process **(**BP**)** (**D**) and KEGG (**E**) pathway enrichment analysis of these CDEGs. **(F)** Lasso regression was performed to develop the optimal model. **G-H.** Distribution of PARS in meta-GEO (**G**) and TCGA-CRC (**H**) cohorts.
